# Quetiapine Ameliorates Schizophrenia-Like Behaviors and Protects Myelin Integrity in Cuprizone Intoxicated Mice: The Involvement of Notch Signaling Pathway

**DOI:** 10.1093/ijnp/pyv088

**Published:** 2015-08-01

**Authors:** Hua-ning Wang, Gao-hua Liu, Rui-guo Zhang, Fen Xue, Di Wu, Yun-chun Chen, Ye Peng, Zheng-wu Peng, Qing-rong Tan

**Affiliations:** Department of Psychiatry, Xijing Hospital, Fourth Military Medical University, Xi’an, China (Drs Wang, Liu, Zhang, Xue, Wu, Chen, Y. Peng, Z.-w. Peng, and Tan); Air Force General Hospital of PLA, Beijing, China (Dr Y. Peng); Department of Toxicology, Shaanxi Key Lab of Free Radical Biology and Medicine, School of Public Health, Fourth Military Medical University, Xi’an, China (Dr Z-w. Peng).

**Keywords:** schizophrenia, quetiapine, myelination, Notch

## Abstract

**Background:**

White matter disturbances and myelin impairment are key features of schizophrenia. The antipsychotic drug quetiapine can promote the maturation of oligodendrocytes, but the molecular mechanisms remain largely unknown.

**Methods:**

The schizophrenia-like behaviors, degrees of demyelination, and levels of Notch signaling molecules in forebrains of adult male C57BL/6 mice were examined after fed with cuprizone (0.2% wt/wt) in the presence or absence of 10mg/kg/d quetiapine for 6 weeks. These parameters were also observed after the transcranial injection of Notch signaling inhibitor MW167 (1mM) daily during the last week of the treatment period.

**Results:**

Quetiapine ameliorated the schizophrenia-like behaviors and decreased expression of myelin basic protein and inhibition of Notch signaling molecules, such as Notch1, Hes1, and Hes5, in the forebrain that induced by cuprizone. These beneficial effects of quetiapine were abolished by MW167.

**Conclusions:**

The antipsychotic and myelin protective effects of quetiapine are mediated by Notch signaling in a mouse model of cuprizone-induced demyelination associated with schizophrenia-like behaviors. The Notch pathway might therefore be a novel target for the development of antipsychotic drugs.

## Introduction

Schizophrenia is a highly debilitating chronic mental disorder that affects about 1% of the population worldwide (Gershon et al., 2011). Schizophrenia comprises complex clinical syndromes that are often divided into positive, negative, and cognitive symptoms. It is thought that environmental and genetic factors combine to cause neurodevelopmental disturbances that lead to schizophrenia in predisposed individuals. However, none of the hypotheses regarding the specific pathogenesis of schizophrenia adequately explain the biological basis of the disorder (Ross et al., 2006).

Recent research implies that white matter pathology is a key feature of schizophrenia (Davis et al., 2003). Functional imaging studies based on diffusion tensor imaging, magnetization transfer ratio, diffusion tensor spectroscopy (Du et al., 2013), and brain positron emission tomography of individuals with schizophrenia have demonstrated myelin pathologies and oligodendrocyte degeneration in many brain regions, including the prefrontal cortex, temporal lobe, corpus callosum, hippocampus, and thalamus of schizophrenia patients (Takahashi et al., 2011). Consistent with these findings, electron microscopy analysis of postmortem samples from patients with schizophrenia indicates that there are aberrant myelination of synaptic terminals, increased density of concentric lamellar bodies, and apoptosis/necrosis of oligodendroglial cells (Miyakawa et al., 1972; Ong and Garey, 1993; Uranova et al., 2001). Furthermore, the expression of important genes for oligodendrocyte development, function, and myelination were found to be abnormal in the schizophrenic brain. Molecular genetics studies verified the abnormal expression of oligodendrocyte/myelin related genes in brain samples of schizophrenia patients (Tkachev et al., 2003; Barley et al., 2009). Taken together, these findings raise the intriguing possibility that aberrations in oligodendrocyte development contribute to schizophrenia pathogenesis, and drugs that reverse abnormal oligodendrocyte phenotypes may be an effective treatment strategy.

The novel second-generation antipsychotic drug quetiapine is commonly used to treat schizophrenia. Quetiapine has a unique receptor binding profile and regulates many genes involved in cell cycle/fate control (Kondo et al., 2013). Recent studies have assessed the effects of quetiapine on demyelination induced by the oligodendrocyte-specific toxin cuprizone. These studies found that quetiapine administration either before or after cuprizone-mediated demyelination significantly enhanced oligodendrocyte regeneration and myelin repair by preserving the number of GST-pi–expressing mature oligodendrocytes, accelerating the maturation of oligodendrocyte precursor cell (OPCs), and promoting the survival of oligodendrocytes (Zhang et al., 2008, 2012). These effects were tightly correlated with an attenuation of pathological behaviors typically induced by cuprizone. However, a more thorough understanding of signaling pathways mediating the pro-oligodendrogliagenic effects of quetiapine may identify novel molecular targets for treating schizophrenia and other mental illnesses.

The Notch pathway has broad roles in cell fate determination and organ formation, including white matter development, and it has been implicated in schizophrenia. Notch signaling is an essential regulator of cell fate specification, asymmetric cell division, and cell morphogenesis in development (see Kopan and Ilagan, 2009 for comprehensive review). In addition, Notch signaling is an indispensable regulator of OPC specification (Artavanis-Tsakonas et al., 1999), oligodendrocyte maturation, and myelin formation (Hu et al., 2003). Thus, Notch signaling in the demyelination/remyelination process will help us further understand demyelinating disorders, such as multiple sclerosis (Jurynczyk and Selmaj, 2010; Stoop et al., 2010; Aparicio et al., 2013). Interestingly, a recent study found there was significant genetic overlap between schizophrenia and multiple sclerosis (Andreassen et al., 2015). Meanwhile, the Notch4 locus has been identified as a candidate susceptibility gene for schizophrenia (Stefansson et al., 2009), and the mRNA level of Notch1 is differentially expressed in parvalbumin-immunoreactive neurons in subjects with schizophrenia (Pietersen et al., 2014). However, the potential relationship between Notch signaling and white matter pathology has not been studied in an animal model of demyelination with behavioral features of schizophrenia; furthermore, it is unclear whether quetiapine affects Notch signaling. Therefore, the primary goal of the present study was to investigate the myelin protection and antipsychotic effects of quetiapine after cuprizone-mediated demyelination and to determine whether these effects were associated with Notch signaling activation in the forebrain of cuprizone-treated and control mice.

## Methods

### Animals and Drug Administration

Adult male C57BL/6 mice (8 weeks old, weighing 18–22g) were used for all experiments and were purchased from the Laboratory Animal Center of the Fourth Military Medical University. Mice were group housed in temperature- and humidity-controlled rooms (22±1°C and 60% humidity) with a 12-hour light/dark cycle. Water and food were available ad libitum throughout the entire experimental period. All animal procedures were performed in accordance with protocols and guidelines approved by the Institutional Animal Care and Use Committee of the Fourth Military Medical University (Xi’an, China).

Cuprizone was mixed with the standard powdered rodent chow at a concentration of 0.2% by weight. Quetiapine was dissolved in distilled water and orally administered at 10mg/kg/d. Normal rodent chow and distilled water served as negative controls. The dosages of cuprizone and quetiapine used in the present study were based on those from Xiao et al. (2008). MW167 was dissolved in saline with 10% dimethyl sulfoxide (DMSO) to the concentration of 1mM (Jurynczyk et al., 2005). Vehicle (saline + 10% DMSO) served as a control treatment. The MW167 and vehicle solutions were injected unilaterally into the right brain lateral ventricle with a Hamilton syringe attached to the double tube trace delivery system at a rate of 0.4 μL/min for 5 minutes. The syringe was kept in place for another 5 minutes to prevent the reflux of the drug.

### Experimental Protocols

#### Experiment 1

**Figure 1.**
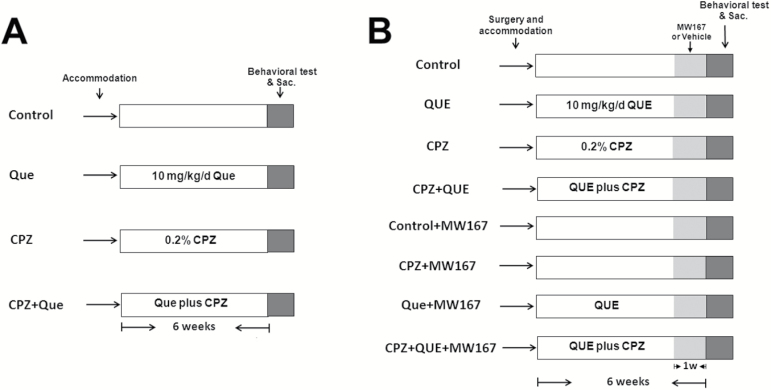
Schematic diagram detailing the time course of cuprizone, quetiapine, and MW167 treatment. (A) The timeline for experiment 1. All animals were subjected to 1 week of adaptation, and then animals were treated with drugs (eg, cuprizone and/or quetiapine) as indicated for 6 weeks, and subjected to 1 week of behavioral analysis before sacrifice. (B) The timeline for experiment 2. This experiment was carried out as in (A), except that mice from all groups went through lateral ventricular catheterization surgery for intracranial delivery of MW167 or vehicle 5 weeks into the 6-week drug treatment. Mice in the control + MW167 and cuprizone + quetiapine + MW167 group were treated with 2 μL of 1mM MW167 daily during the final week of the drug treatment period. At the end of the experiments described in (A) and (B), animals were sacrificed to assess molecular differences among all groups.

The cuprizone-mediated animal model of demyelination with behavioral features of schizophrenia was carried out according to a procedure previously described (Xiao et al., 2008) with modifications. Mice (n=48) were randomly distributed into 4 groups (12/group): Control group, quetiapine (Que) group, cuprizone (CPZ) group, and cuprizone + quetiapine (CPZ + Que) group. The detailed experimental procedures are shown in Figure 1. After a 1-week accommodation period, the mice received the following treatments: CPZ group mice were administered 0.2% by weight cuprizone in standard powdered rodent chow for 6 weeks; concurrently, the control group mice were fed a normal powdered rodent chow. The Que group mice received normal powdered rodent chow plus quetiapine dissolved in distilled water (10mg/kg/d) for 6 weeks, and the CPZ + Que group mice were fed 0.2% by weight of cuprizone and 10mg/kg/d quetiapine. Twenty-four hours after the completion of the treatment period, all mice were subjected to prepulse inhibition, open field, Y-maze, and 3-chamber tests. Then the same mice that underwent behavioral testing were randomly divided into 2 subgroups: mice of subgroup 1 (n=6) were used for immunofluorescent staining and mice from the subgroup 2 (n=6) were anaesthetized with chloral hydrate and their brains quickly dissected on ice that were then used for Western blot and real-time polymerase chain reaction (RT-PCR) assays.

#### Experiment 2

In this experiment, we tested the effect of γ-secretase inhibition with MW167 on quetiapine modulation of schizophrenia-like behaviors. Animals were randomly divided into 8 groups (n=12 for each group): control, Que, CPZ, CPZ + Que, control + MW167, Que + MW167, CPZ + MW167, and CPZ + Que +MW167 group. Mice underwent right lateral ventricular catheter surgery to deliver the γ-secretase inhibitor MW167 or vehicle and were left to recover for 1 week. Cuprizone (0.2 % by weight) and quetiapine (10mg/kg/d) were administered for 6 weeks. Vehicle and 2 μL of 1mM MW167 were injected intraventricularly daily during the final week of the cuprizone/quetiapine administration period. All mice were subjected to neuropsychiatric behavioral tests 24 hours after treatment. Then mice of each group were randomly divided into 2 subgroups, as in experiment 1.

### Drugs and Antibodies

Mouse anti-CNPase, rabbit anti-Hes1, and rabbit anti-Hes5 antibodies were purchased from Abcam Corporation (Cambridge, UK). Anti-MBP antibodys were obtained from Millipore Corporation (Billerica, MA) for immune staining or ABcam (ab62631) for Western blot, and immobilon Western chemiluminescent HRP substrate were obtained from Millipore Corporation. Mouse anti-β-actin monoclonal antibody was purchased from CWBIO Corporation (Beijing, China). HRP-linked anti-rabbit and anti-mouse IgG antibodies were from Cell Signaling Corporation (Beverly, MA). Alexa Fluor 594 Donkey anti-rat IgG was from Invitrogen Corporation (San Diego, CA), and primers of *Notch1*, *Notch2*, *Notch4*, *Hes1*, and *Hes5* were synthesized by TaKaRa Corporation (Seta, Japan). PrimeScript RT reagent Kit with gDNA Eraser, SYBR Premix Ex Taq (TliRNaseH Plus), and EASY Dilution were also from TaKaRa Corporation. Cuprizone (bis-cyclohexanoneoxalydihydrazone, CPZ) was purchased from Sigma Corporation (Ronkonkoma, NY). Quetiapine was a generous gift from Professor Li Xinmin, University of Manitoba, Canada. Halothane was obtained from Halocarbon Laboratories (Kinderkamack, NY). Biodentine dental cement was obtained from Septodont Corporation (Saint Maur des Fossés, France). DMSO was obtained from Sigma Corporation, and MW167, a γ-secretase II inhibitor, was purchased from Calbiochem Corporation (La Jolla, CA).

### Intracerebroventricular Injections

To deliver MW167 intracerebrally, the protocol for intracerebroventricular injection was adopted from Yanamandra et al. (2013), and the coordinates were opted referring to the mouse brain in stereotaxic coordinates. Mice were deep anaesthetized with chloral hydrate. When the needle was positioned directly over the bregma point, the *x* and *y* coordinates were set to zero. A central transcranial hole (about 1mm in depth) was made through the skull at *x*, -0.7mm and *y*, 0.5mm for the insertion of the needle. Three other small holes were drilled around the central one, and into each one, a steel screw was fixed. When the needle touched the endocranium, the *z* coordinate was set to zero. Then the needle was slowly inserted into the right ventricle to a depth of 2.0mm below the surface of the endocranium. The biodentine bioactive and biocompatible dentin substitute (Septodont Corporation) were used to cover and fasten the outer part of the trace administration system. Finally, mice were kept on 37°C thermostat boards until revival.

### Behavioral Tests

The method of the open field test was adopted from Masato Fukui et al. (2007) with some adaptations. Briefly, all mice were moved to the experimental room and adapted for 30 minutes before each experiment. Each mouse was put in the middle of the open field apparatus (25 cm×25 cm×45cm, 340 lux), and we recorded the time, distance (mm), and trajectory every 15 minutes. A camera hanging on top of the open field apparatus was used.

The Y-maze apparatus used in our experiment was composed of 3 arms (A, B, and C) positioned at an equal angle (120°) and was surrounded by various extra-maze cues. Each arm was 30cm long, 10cm wide, and 45cm above the ground. Each mouse was randomly put in 1 arm and allowed to explore all 3 arms freely for 8 minutes. The apparatus was wiped thoroughly with 75% ethanol between subjects to prevent odor interference and cross infection. The order of the exploration of each arm was recorded manually, and the entire exploration process was recorded by a video camera for later analysis and confirmation. An alternation was defined as entries into all 3 arms on consecutive occasions. Therefore, the maximum alternation was the total number of arm entries minus 2, and the percentage of alternation was calculated as (actual alternations/maximum alternations) × 100.

The prepulse inhibitions (PPIs) were measured by exposing the mice to a series of acoustic pulses with or without a short acoustic prepulse as previously described (Yang et al., 2015). An animal acoustic startle system (Coulbourn Instruments) was used for testing. Briefly, mice were housed in a sound-attenuated room with a 65-dB background noise. After a habituation period of 5 minutes, 74 trials were conducted in each test session, with an average inter-trial interval of 15 seconds. The first and last 12 trials (Blocks 1 and 3) each consisted of a single 40-ms, 120-dB startle stimulus. The middle 50 trials (Block 2) consisted of random delivery of 10 trials of startle stimulus alone, 10 no-stimulus trials, and 30 prepulse trials. The prepulse trials consisted of a single 120-dB startle stimulus preceded by a 20-ms nonstartling prepulse stimulus of 3, 6, or 12 dB above the background noise. The PPI score was calculated using the data of Block 2 with the following formula: [1 − (startle amplitude following prepulse + pulse pair/startle amplitude following pulse-alone)] × 100%.

The 3-chamber test was adopted from a previously published protocol (Kaidanovich-Beilin et al., 2011). The apparatus was a rectangular box composed of 3 connected chambers of the same size (30 cm×30 cm×30cm). A wire-cup like container with a removable lid that was large enough to hold a single mouse was fixed in the middle of each side chamber. The experiment consisted of 2 sessions. In session 1, one control mouse (Stranger 1) of the same background, age, gender, and weight as the subject mouse was put inside a wire cup-like container. The subject mouse was put in the middle chamber for 5 minutes before being given access to side chambers for 10 minutes. The time subject mice spent in each chamber was recorded with a suspended video camera. In session 2, another unfamiliar mouse (Stranger 2, also of the same background, age, gender, and weight) was put in the previously empty container. The time spent in each chamber was then calculated. Note that stranger mice had no encounters with the subject mice prior to testing.

### Immunofluorescent Staining For MBP

Twenty-four hours after the behavioral tests, one-half of the mice from each group were deeply anaesthetized with chloral hydrate and perfused through the ascending aorta with 0.1M phosphate buffered saline (PBS, pH=7.4), followed by 4% paraformaldehyde in PBS. Their brains were carefully removed, post-fixed in the same solution for 1 hour, and then dehydrated in 25% sucrose in PBS at 4°C for 24 to 48 hours. Serial coronal sections (20 μm) of the brains were cut using a sliding microtome (Leica CM3050 S, Buffalo Grove, IL) and collected in 6-well plates containing 0.01M PBS. Three sets of sections were used for immunofluorescence staining. Briefly, free-floating sections were incubated for 24 hours at room temperature with a primary antibody to MBP (1:400, rat anti-MBP antibody, Millipore Corporation) in blocking solution composed of 0.2% Triton X-100 and 5% bovine serum albumin (Sigma Corporation, St. Louis, MO) in PBS. These sections were then incubated with Alexa Fluor 594 Donkey Anti-rat IgG secondary antibody (Invitrogen Corporation, San Diego, CA) at room temperature for 3 hours. Sections with the primary antibody omitted were used as negative controls.

### Western Blotting

Mice forebrain tissues were collected and lysed with a protein extraction kit (KeyGEN BioTECH, Nanjing, China). Then, 40 μg of total protein was resolved on 8% sodium dodecyl sulfate polyacrylamide gel electrophoresis gels and transferred onto polyvinylidene fluoride membranes. The membranes were blocked with 5% nonfat milk in PBST before being blotted with antibodies against MBP, Hes1, and Hes5 at 4°C overnight. The membranes were washed and incubated with appropriate HRP-conjugated secondary antibodies. Specific protein bands were visualized using the immobilon Western chemiluminescent HRP Substrate (Millipore Corporation) and analyzed with the Image J software.

### Quantitative RT-PCR

The forebrains were homogenized in Trizol reagent (Invitrogen Corporation) and extracted with chloroform on ice. The RNA pellets were precipitated with isopropanol, washed with precooled 75% ethanol, and dissolved in 10 μL DEPC-treated water and an aliquot was used for determination of the amount of RNA. Reverse transcription was performed using the PrimeScript TM RT reagent kit with gDNA Eraser (TaKaRa Corporation) according to the manufacturer’s protocol with 1 µg of total RNA template. Quantitative RT-PCR was performed with SYBR Premix Ex TaqTM (TliRNaseH Plus) (TaKaRa Corporation) and detected by the CFX96 Real-Time PCR Detection System (Bio-Rad Corporation, Hercules, CA). The sequences for RT-PCR primers are listed in Table 1. The relative gene expression level was calculated by the 2-ΔΔCt method with *gapdh* used as the internal control.

**Table 1.** Primers for RT-PCR

Gene	Forward	Reverse
*Notch2*	5′-ACTGGGCAGCTGCTGTCAATAA	5′-AAGGCGGTCCATGTGGTCA
*Notch4*	5′-CAGGATCCACCTGTCACCAAGA	5′-CGTGTAACCAGGCAGGCAGA
*Notch1*	5′-GCGAAGTGGACATTGACGAG	5′-GCTGGCACAGGCAGGTAAAG
*Hes1*	5′-GCAGACATTCTGGAAATGACTGTGA	5′-GAGTGCGCACCTCGGTGTTA
*Hes5*	5’-AGTCCCAAGGAGAAAAACCGA	5’-GCTGTGTTTCAGGTAGCTGAC
*gapdh*	5’-GATTCGGGCCACTTGGAGTTA	5’-TGGAGCAACACCAGGCAGAC

### Statistical Analysis

Statistical analyses were performed using SPSS 19.0. All values are presented as the means±SEM. Data were subjected to 1-way or 2-way ANOVA followed by LSD-t or Student–Newman–Keuls posthoc tests for between-group comparisons. To detect different manifestations of social behaviors between each experimental group, paired t tests were used to compare sets of 2 groups. Differences were considered significant when *P*<.05.

## Results

### Quetiapine Reduced Cuprizone-Induced Schizophrenia-Like Behaviors

**Figure 2.**
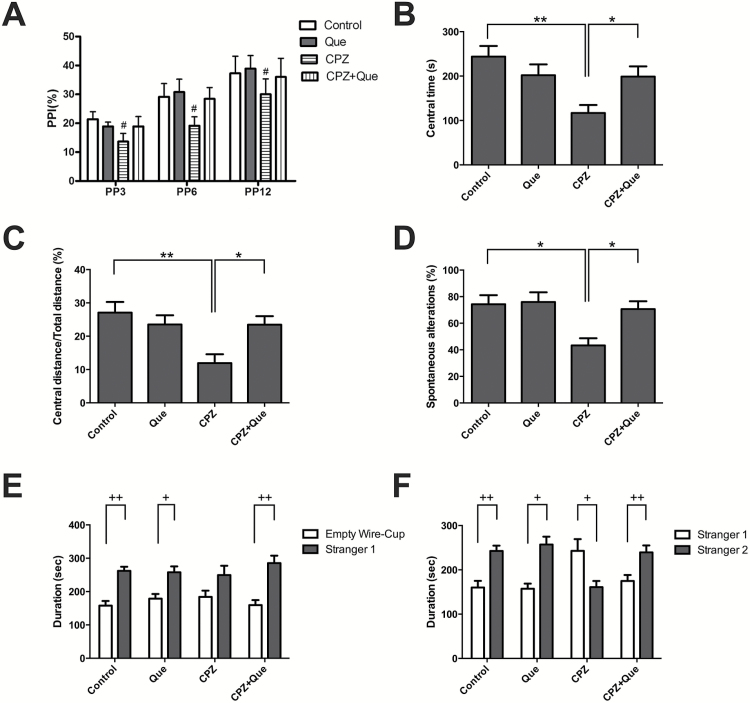
Behavioral effects of cuprizone and quetiapine treatment. (A) The cuprizone-treated mice had an inferior prepulse inhibition (PPI), which was reversed by quetiapine treatment (main effect of treatment, *F*_3, 92_=3.835, *P*=.013). (B-C) The cuprizone-treated mice spent less time (*F*_3, 20_=4.886, *P<*.05), travelled a shorter distance (*F*_3, 18_=4.212, *P<*.05) in the central area of the open field test, and exhibited anxiety-like behaviors, which were ameliorated by quetiapine cotreatment. (D) In the Y-maze test, cuprizone-treated mice showed impaired spatial working memory and spatial cognitive ability, which was rescued by quetiapine administration (*F*_3, 25_=3.114, *P<*.05). (E-F) In the 3-chamber test, mice in the cuprizone group showed reduced social interactions and weaker social memory. Mice in the cuprizone + quetiapine group performed better than those treated with cuprizone alone. #*P<*.05 compared with the control group or the CPZ + Que group; *P<.05, **P<.01, ^+^ *P<*.05, ^++^ *P<*.01, compared with the empty wire cup or Stranger 1.

To determine the effects of quetiapine on cuprizone-induced schizophrenia-like behaviors, we carried out a battery of behavioral assays comprised of the PPI test, open field, Y-maze, and 3-chamber tests. In the PPI test, the mice in CPZ group had an inferior PPI compared with the control (Figure 2A). In the open field test, mice treated with cuprizone displayed a reduced central area staying time (116.92±39.98 s; Figure 2B) and a reduced percentage of central area distance traveled/ total distance (11.90%±5.34%; Figure 2C) compared with the control group (*P*<.05). Quetiapine relieved all the aforementioned effects of cuprizone on mice (Figure 2A-C). In addition, cuprizone-treated mice showed impaired spatial cognitive ability, indicated by a significant decrease in alteration behavior in the Y-maze test (50.44%±11.91%), compared with the control group (76.41%±4.48%). This deficit was rescued by quetiapine treatment (Figure 2D).

The 3-chamber test was then used to assess sociability and interest in social novelty. Cuprizone suppressed social affiliation and social memory compared with control mice, and this was rescued by quetiapine treatment (Figure 2E-F). Specifically, in session 1, control mice spent more time in the compartment with Stranger 1 (262.01±39.62 s) than in the compartment with the empty-wire cup (147.72±37.38 s, *P*<.01), while cuprizone-treated mice spent a similar amount of time in both chambers (249.51±62.92 s vs 184.21±41.36 s, *P* = 0.088). In contrast, quetiapine treatment resulted in the cuprizone-treated mice spending more time in the compartment with Stranger 1 (257.93±46.09 s vs 164.56±16.38 s, *P<*.01). In session 2, when a novel unfamiliar mouse (Stranger 2) was put in the previously empty cup, control mice spent more time with Stranger 2 (242.68±37.48 s) than with Stranger 1 (160.00±23.96 s, *P<*.01), whereas mice treated with cuprizone spent much less time with Stranger 2 (160.85±31.30 s vs 242.83±59.55 s, *P<*.05). Cuprizone-treated mice spent more time with Stranger 2 if they were administered quetiapine (239.31±47.73 s vs 174.81±40.74 s, *P<*.01) (Figure 2F).

### Quetiapine Inhibited Cuprizone-Induced Demyelination

**Figure 3.**
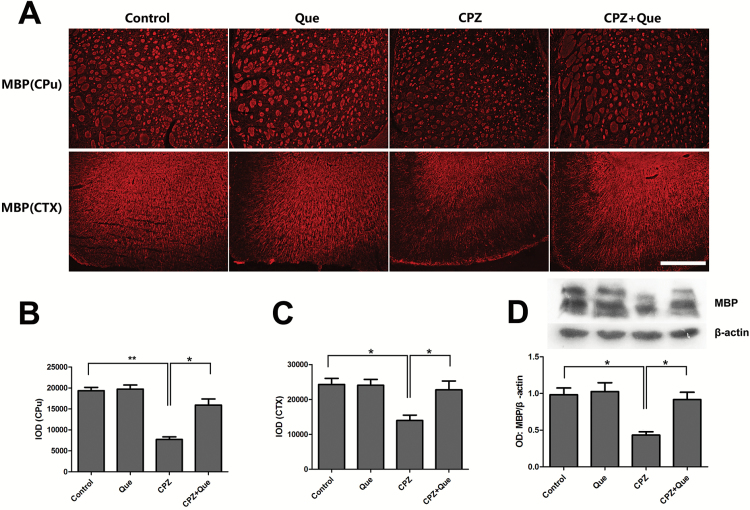
Quetiapine protected the forebrain from demyelination. (A) MBP immunofluorescent in the ventral lateral part of the caudoputamen (CPu) and cerebral cortex (CTX) of mice in each group (n=6). (B-C) Histogram represents the quantitative analysis of the integral optical density (IOD) of MBP level in CPu (*F*_3, 20_ = 33.79, *P<*.01) and CTX (*F*_3, 20_=5.978, *P<*.01). (D) Western-blot assays demonstrated reduced expression of MBP in the forebrain of cuprizone-treated mice was restored by quetiapine cotreatment (*F*_3, 20_=7.084, *P<*.01). Scale bar A=500 μm. *P<.05, **P<.01.

We then determined if quetiapine had rescued cuprizone-induced demyelination by immunostaining and Western-blot analysis of myelin and MBP in the forebrain. MBP was downregulated in the forebrains of cuprizone-treated mice (Figure 3). Immunofluorescent staining showed a loss of MBP in the lateral ventral part of the caudoputamen (CPu) and the cerebral cortex (CTX) and restorations of MBP towards control levels in the lateral ventral part of the CPu and the CTX in the cuprizone + quetiapine group. Western blot results showed a significant increase of MBP expression in cuprizone + quetiapine-treated mice compared with cuprizone only-treated mice.

### Quetiapine Activated Notch Signaling

**Figure 4.**
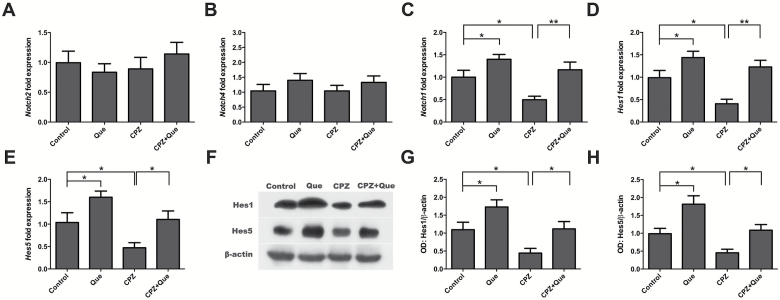
Examination of the expression of molecules in the Notch signaling pathway in the forebrain of mice for all treatment groups (n=6 for each group). (A-B) RT-PCR showed no change of *Notch2* (*F*_3, 25_=0.323, *P*=.809), and *Notch4* levels (*F*_3, 18_ = 0.708, *P*=.56) in each group. (C-E) RT-PCR results demonstrated that *Notch1*, *Hes1*, and *Hes5* were upregulated by quetiapine and downregulated by cuprizone. *Notch1* (C) (*F*_3, 24_ = 8.335, *P<*.01), *Hes1* (D) (*F*_3, 22_ = 8.637, *P<*.01), and *Hes5* (E) (*F*_3, 24_ = 4.478, *P<*.05) all increased in the cuprizone/quetiapine cotreatment group. (F-H) Western-blot analysis showed decreased expressions of Hes1 (*F*_3,22_=7.498, *P<*.01), and Hes5 (*F*_3, 22_=10.335, *P<*.01) in CPZ group and were upregulated after quetiapine co-administration. *P<.05, **P<.01.

To determine if quetiapine regulates the Notch pathway, we then assessed the expression levels of several Notch receptors and the bHLH transcription factors Hes1 and Hes5, which are targets of Notch signaling. Although *Notch2* and *Notch4* mRNA levels were not affected, *Notch1*, *Hes1*, and *Hes5* expression levels were all reduced by cuprizone in the mouse forebrain, and this deficit was reversed by quetiapine administration (Figure 4A-E). Similar results were observed at the protein level by Western blotting for Hes1 and Hes5 (Figure 4F-H).

### MW167 Inhibited the Protective Effects of Quetiapine against Cuprizone-Induced Schizophrenia-Like Behaviors

**Figure 5.**
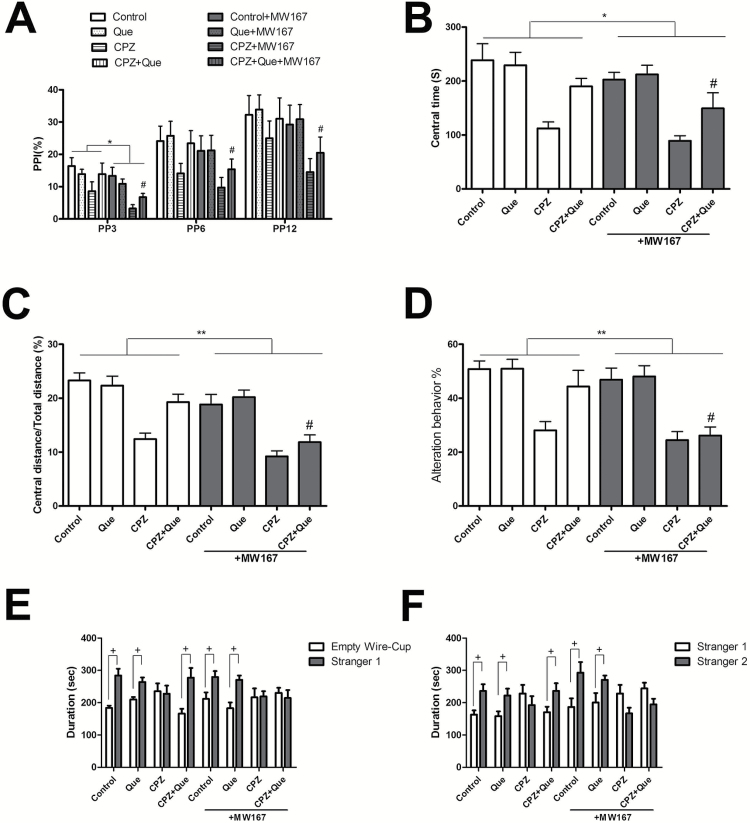
The antipsychotic effects of quetiapine were inhibited by MW167. (A) MW167 could affect the prepulse inhibition (PPI) and block the increased PPI induced by quetiapine. (B-C) The open field test revealed the anti-anxiety effects of quetiapine were blocked by MW167. (D) The Y-maze test showed quetiapine rescue of working memory deficits was blocked by MW167 injection. (E-F) In the 3-chamber test, mice in the cuprizone (CPZ) group and the CPZ + quetiapine (Que) + MW167 group showed reduced social interactions and weaker social memory. Mice in the cuprizone + quetiapine group performed better than those treated with cuprizone or cuprizone plus MW167. *P<.05, **P<.01; ^+^ *P<*.05, compared with the empty wire-cup or Stranger 1; # *P<*.05 compared with CPZ + Que group.

To determine if Notch signaling is necessary for quetiapine-mediated effects on myelination and behavior, we blocked Notch activity in the forebrain by injecting the γ-secretase inhibitor MW167 into the right lateral ventricle during the last week of cuprizone treatment. Specifically, 2-way ANOVA revealed significant differences of main effect of treatment (control vs Que vs CPZ vs CPZ + Que) in PPI at 3 dB (*F*_3, 56_ = 5.598, *P<*.01) or 6 dB (*F*_3, 56_ = 3.342, *P* = 0.023) above the background noise, central time (*F*_3, 56_ = 15.814, *P<*.01) and the percentage of central distance/total distance (*F*_3, 56_ = 24.295, *P<*.01) in the open field test, and the percentage of alteration behavior (*F*_3, 56_=16.702, *P<*.01) in the Y-maze test. There were also significant differences of MW167 treatment effect (MW167 vs vehicle) in PPI at 3 dB (*F* = 8.365, *P<*.01) above the background noise, central time (*F* = 4.167, *P* = 0.046) and the percentage of central distance/total distance (*F*=17.970, *P<*.01) in the open field test, and the percentage of alteration behavior (*F*=6.788, *P*=.012) in the Y-maze test. Additionally, posthoc comparisons showed that there were no significant differences between CPZ and CPZ + Que +MW167 in the above behavioral parameters. Indicating MW167 administration could affect the behavior of mice and inhibit the PPI improvement, anxiolytic, spatial memory restoring, and elevated interest in social novelty of quetiapine (Figure 5). In session 1 of the 3-chamber assay, mice form control, Que, and CPZ + Que groups spent a significantly longer time in the compartment with Stranger 1 than in the compartment holding the empty-wire cup, and mice from control + MW167 and Que + MW167 groups also spent a significantly longer time in the compartment with Stranger 1 than in the compartment holding the empty-wire cup. Notably, mice in the CPZ + MW167 and CPZ + Que + MW167 groups spent nearly the same length of time in the compartment with Stranger 1 as they did in the compartment with the empty-wire cup as well as the appearance of mice in CPZ group (Figure 5E). In session 2, mice from control, CPZ + Que, control + MW167, and Que + MW167 groups spent a significantly longer time in the compartment with Stranger 2 than in the compartment with Stranger 1 (*P<*.05). Mice from CPZ + MW167 and CPZ + Que + MW167 groups spent a similar amount of time in the compartment with Stranger 2 as in the compartment with Stranger 1 (*P* > 0.05), similarly with the appearance of mice in CPZ group (Figure 5F).

### MW167 Inhibited the Pro-Myelinating Effects of Quetiapine

**Figure 6.**
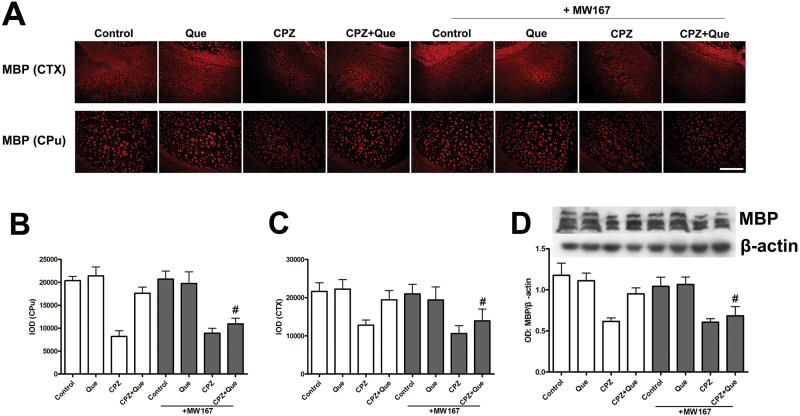
The pro-myelinating effects of quetiapine require Notch signaling activation. Microphotographs (A) and histograms (B-C) represent the expression and quantitative analysis of the integral optical density (IOD) of MBP level. Cuprizone induced significant demyelination in the cerebral cortex (CTX) and ventral lateral caudoputamen (CPu), which was reversed by quetiapine coadministration. Intra-ventricular administration of MW167 reduced the myelin-protective effect of quetiapine. (B) Western-blot assays illustrating reduced expression levels of MBP induced by cuprizone in the mouse forebrain. MBP was upregulated by quetiapine cotreatment and this effect was blocked by MW167. Scale bar A=500 μm. #*P<*.05 compared with CPZ + Que group.

Two-way ANOVA revealed significant differences of main effect of treatment in integral optical density (IOD) of MBP level in CPu (*F*_3, 40_ = 26.402, *P<*.01) or CTX (*F*_3, 40_ = 6.286, *P<*.01) as well as the expression of MBP in the forebrain (*F*_3, 32_ = 12.374, *P<*.01). There were no significant differences of MW167 treatment effect in IOD of MBP level in CPu (*F* = 2.649, *P* = 0.111) or CTX (*F* = 2.467, *P* = 0.124) and the expression of MBP in the forebrain (*F* = 1.575, *P* = 0.219). Additionally, posthoc comparisons showed that there were significant differences between CPZ+ Que and CPZ + Que +MW167 in these parameters, and there were no significant differences between CPZ and CPZ + Que +MW167 in these parameters. Indicating MW167 administration could inhibit the myelin protective effect of quetiapine (Figure 6).

### MW167 Inhibited the Expression of Notch1 Signaling Molecules in Cuprizone- and Quetiapine-Treated Mice

**Figure 7.**
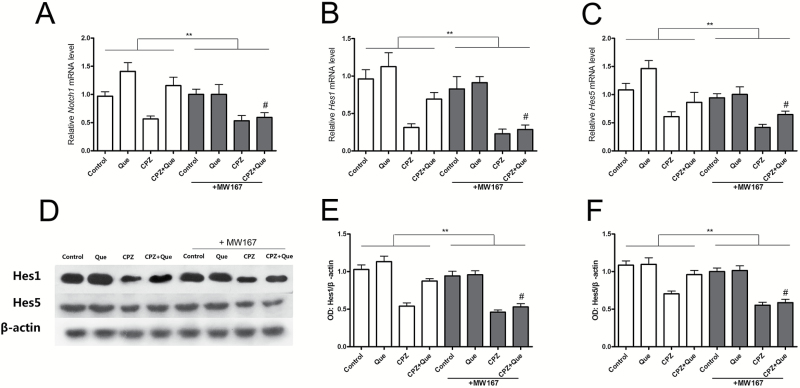
Examination of the expression of molecules in the Notch signaling pathway in the forebrain of mice after MW167 and quetiapine treatment. (A-C) RT-PCR showed that the elevated mRNA levels of *Notch1*, *Hes1*, and *Hes5*of quetiapine were inhibited by MW167. (D-H) Western-blot analysis showed the rescue expressions of Hes1 and Hes5 in the quetiapine-treated group were blocked by MW167. **P<.01, #*P<*.05 compared with CPZ + Que group.

As shown in Figure 7, 2-way ANOVA revealed significant differences of main effect of treatment in the mRNA levels of *Notch1* (*F*_3, 30_ = 9.842, *P<*.01), *Hes1* (*F*_3, 28_ = 23.16, *P<*.01), and *Hes5* (*F*_3, 28_ = 14.321, *P<*.01), and there were also significant differences of MW167 treatment effect in mRNA levels of *Notch1* (*F* = 7.918, *P<*.01), *Hes1* (*F* = 8.091, *P<*.01), and *Hes5* (*F* = 9.501, *P<*.01). Consistent with these data, there were significant differences of main effect of treatment (*F*_3, 32_=47.570, *P<*.01; *F*_3, 32_=28.270, *P<*.01) and MW167 treatment effect (*F*=21.459, *P<*.01; *F*=19.217, *P<*.01) in the protein expression of Hes1 and Hes5. Additionally, posthoc comparisons showed that there were significant differences between CPZ+ Que and CPZ + Que +MW167 in these parameters, and there were no significant differences between CPZ and CPZ + Que +MW167 in these parameters.

## Discussion

In the present study, it was found that quetiapine, a unique APD, exerted its antipsychotic and myelin protective effect in a Notch-dependent manner. It is well documented that cuprizone impairs myelin integrity and induces schizophrenia-like behaviors due to its toxicity to oligodendrocytes (Gregg et al., 2009). Our results similarly demonstrated that cuprizone led to damage of myelin sheaths in the mouse forebrain. This damage to myelin was associated with downregulation of the expression of molecules in the Notch signaling pathway, the induction of anxiety-like behaviors, and impaired social and spatial cognition. Coadministration of quetiapine restored myelin formation, stimulated the expression of Notch molecules, and alleviated the above behavior changes induced by cuprizone treatment. Thus, our findings indicate that quetiapine may exert its therapeutic effects at least in part by reversing abnormalities of myelination through Notch signaling.

During the past 10 years, evidence obtained through imaging (Ellison-Wright et al., 2008; Glahn et al., 2008), genetic (Hakak et al., 2001; Tkachev et al., 2003; Katsel et al., 2005), and postmortem (Hof et al., 2003; Stark et al., 2004; Uranova et al., 2004) studies indicate that white matter pathology plays an important role in the pathogenesis of schizophrenia. Moreover, psychosis is observed in patients with leukoencephalopathy (Denier et al., 2007) and multiple sclerosis (Patten et al., 2005). APDs alleviate psychotic symptoms and restore myelin integrity in both schizophrenia (Garver et al., 2008) and MS (Zhornitsky et al., 2013). Changes in the levels of mRNA transcripts for myelin genes have been observed in the absence of changes in neuronal genes in brain tissue from individuals with schizophrenia, suggesting that changes in myelin gene expression and white matter structure may be a direct cause of schizophrenia. This implies that drugs impacting the transcription of myelin genes may relieve psychotic symptoms and improve disease outcome. Thus, our findings are in keeping with increasing evidence of oligodendrocyte and myelin abnormalities. Moreover, they demonstrate that drugs such as quetiapine rescue myelin phenotypes are effective treatments for schizophrenia.

In addition to their well-defined role in myelination, oligodendrocytes have a number of other important biological functions. Oligodendrocytes not only regulate the microenvironment around neurons (Ludwin, 1997) but also secrete trophic factors that are critical for the survival of neurons and astrocytes (Espinosa de los Monteros et al., 1989). Oligodendrocyte development dysregulation and the resultant malformation of myelin may result in axon degeneration and subsequent neuron death. Myelin defects may thereby disturb the synchronization between different brain regions. Human brains constantly undergo myelin/oligodendrocyte renewal through life (Paus et al., 1999; Dimou et al., 2008; Rivers et al., 2008; Young et al., 2013). Thus, disruption of myelin remodeling and plasticity could cause a number of psychiatric disorders in addition to schizophrenia. As a result, abnormalities in oligodendrocytes may have far-reaching consequences on neurological function and behavior. Determining the molecular mechanisms by which oligodendrocytes regulate these processes and how they go wrong in disease is essential for developing improved therapies.

Our study indicates that quetiapine may be particularly effective in reversing the PPI inferior, anxiety, impaired spatial memory, and interest in social novelty in CPZ-treated mice. It is important to note that anxiety and depression are the most frequently reported early signs and symptoms of schizophrenia, often occurring before the first psychotic episode (Iyer et al., 2008). These symptoms represent important information in the diagnostic process. In addition, individuals with schizophrenia often have primary motor dysfunctions, including hypokinesia and retarded catatonia, which are closely related to negative and disorganized symptoms (Peralta et al., 2010).

Dysmyelination in the PFC may also account for disturbances of spatial working memory in schizophrenia (Piskulic et al., 2007; Schlosser et al., 2007). Y-maze test performance, which is a working memory test, is highly correlated with oligodendrocyte function (Zhang et al., 2012). In our study and others, the cuprizone model has been shown to induce disturbance in spatial cognition caused by oligodendrocyte malfunction, impaired oligodendrocyte regeneration, and obstruction of oligodendrogliagenesis. In animal models, negative symptoms are analyzed by the social interaction test (Sandhu et al., 2014) and result from, at least in part, the dysgenesis of myelin sheath (Xu et al., 2010). Quetiapine not only ameliorates negative symptoms in parallel with cognitive malfunctions of schizophrenia but also improves emotional symptoms of other psychiatric disorders, such as depression and bipolar disorder (Rowe, 2007; Sanford and Keating, 2012). The results of our study and others indicate that quetiapine has protective effects on myelin and oligodendrocytes regulatory activities (Zhornitsky et al., 2013), which may underlie its ability to ameliorate cognitive abnormalities. Notably, there is no significant difference in Notch4 transcription between CPZ-treated mice and control in the forebrain, which has been established as a schizophrenia locus in the previous studies, suggesting that a CPZ-induced demyelination animal model could mimic part of the symptoms and not fully explain the mechanisms of schizophrenia.

We have presented novel findings that quetiapine acts through the Notch pathway to protect the integrity of myelin. We found that cuprizone administration led to downregulation of the expression of the Notch1 receptor,and the Hes1 and Hes5 transcriptional regulators, while quetiapine upregulated their transcription. To further determine if Notch signaling modulation was necessary for the effects of quetiapine and cuprizone on schizophrenia and myelination, MW167 was administrated intraventricularly to block the activation of Notch signaling. We found that MW167 inhibited the protective effect of quetiapine from inducing increased expression of Notch signaling pathway proteins and mRNA. These results indicated that Notch signaling might be responsible for mediating the remyelination-dependent antipsychotic action of quetiapine in the forebrain.

Notch has well-described roles in development and promotes the production of glia, including oligodendrocytes. Notch1, Hes1, and Hes5 are all important cell fate-controlling molecules in the CNS (Ohtsuka T, 1999; Yoon and Gaiano, 2005). Notch1 has been found to be an indispensable initiator of the specification and maturation of glial cells (Morrison et al., 2000). For instance, constitutive activation of Notch1 has been shown to block neuronal differentiation (Coffman et al., 1993) and promote the formation of OPCs (Zhou et al., 2001). Quetiapine has been shown to regulate the cell cycle of OPCs and neurons in the prefrontal cortex (Kondo et al., 2013), promote OPC proliferation, oligodendrocyte differentiation, and myelin formation (Zhang et al., 2012). Thus, quetiapine may ameliorate spatial cognition and social interaction in cuprizone-treated mice via Notch-dependent myelin protective effects.

In conclusion, we used cuprizone-induced demyelination as an animal model of schizophrenia to investigate the mechanisms underlying the antipsychotic effects of quetiapine, and demonstrated that quetiapine could alleviate schizophrenia-like behaviors induced by cuprizone and promote remyelination in the forebrain by activating the Notch pathway. Although the CPZ-fed mice could be considered as an animal model to explore roles of white matter abnormalities in the pathophysiology and treatment of schizophrenia, it could not explain the pathogenesis of schizophrenia about genetic and neurotransmitter abnormity. Meanwhile, the multiple testing methods in the present study are explorative, and that may lead to false positive results. Extensive studies are necessary to further address the detailed Notch signaling cascades involved in the pathophysiology of schizophrenia and the pharmacological actions of quetiapine.

## Statement of Interest

All authors report no biomedical financial interests or potential conflicts of interest.
